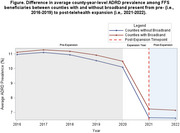# Effect of the 2020 CMS telehealth expansion on diagnostic prevalence of Alzheimer's disease and related dementias

**DOI:** 10.1002/alz70860_105857

**Published:** 2025-12-23

**Authors:** Jenna Rajczyk, James Burke, Jeffrey Wing

**Affiliations:** ^1^ Ohio State University, Columbus, OH, USA

## Abstract

**Background:**

With population aging, underdiagnosis of Alzheimer's disease and related dementias (ADRD) may result to missed opportunities to improve health and may stem from insufficient diagnostic service availability. Telehealth, which relies on the presence of area‐level broadband access, introduces opportunities to improve access to ADRD diagnostic services, such as cognitive assessments (covered in the 2020 Centers for Medicare and Medicaid Services (CMS) telehealth expansion). We aim to investigate the effect of this expansion among fee‐for‐service (FFS) beneficiaries on county‐level prevalence of ADRD diagnoses.

**Method:**

A county‐level ecologic difference‐in‐difference (DID) design was employed to investigate the change in ADRD prevalence among US 65+ FFS beneficiaries from pre‐expansion (i.e., 2016‐2017) to post‐expansion (i.e., 2021‐2022) by comparing counties with and without broadband. County‐year‐level ADRD prevalence was estimated using 2016‐2022 prevalence estimates defined by Chronic Conditions Data Warehouse methodology from the CMS Mapping Medicare Disparities Tool. County‐year‐level broadband presence was defined as counties with 1 high‐speed internet provider and average download/upload speeds of 25/3 megabits‐per‐second (mbps) (Federal Communications Commission Broadband Deployment Data). The parallel trends assumption was assessed using graphical and model‐based approaches. Negative binomial regression models estimated the average effect of the 2020 CMS expansion on ADRD prevalence among counties with broadband. Effect measure modification (EMM) by rural/urban classification (Rural‐Urban Continuum Codes) was evaluated.

**Result:**

Average county‐level ADRD prevalence was 11.06% across the pre‐expansion period and 7.15% post‐expansion. Broadband was present in 70.35% of counties pre‐expansion and 91.96% of counties post‐expansion. Differences in ADRD prevalence by broadband were constant across the pre‐expansion period (Figure). ADRD prevalence was 2.48% higher in counties with broadband versus counties without broadband post‐expansion compared to pre‐expansion (95% Confidence Interval (CI): 1.01, 1.04; Figure). EMM was not present by rurality (p_int_=0.8063).

**Conclusion:**

Preliminary evidence supports a possible positive relative effect of the CMS telehealth expansion on ADRD diagnostic prevalence among the FFS 65+ population in counties with broadband. While improvements in ADRD detection may have occurred in counties with broadband following the expansion, the substantial decrease in ADRD prevalence overall is likely a consequence of the COVID‐19 pandemic and should be explored further.